# Early-Onset Oral Tongue Squamous Cell Carcinoma in the Absence of Traditional Risk Factors: A Case Report with Whole-Exome Sequencing Analysis

**DOI:** 10.3390/reports9020130

**Published:** 2026-04-24

**Authors:** Evgeniy Aleksiev, Darina Lyudmilova Kachakova-Yordanova, Vanyo Mitev, Martin Marinov Georgiev, Zornitsa Mihaylova

**Affiliations:** 1 Research Institute of Innovative Medical Science, Medical University Sofia, Akad. Ivan Geshov 15, 1431 Sofia, Bulgaria; e.petkov@fdm.mu-sofia.bg (E.A.); vmitev@mu-sofia.bg (V.M.); 2 Department of Dental, Oral and Maxillofacial Surgery, Faculty of Dental Medicine, Medical University Sofia, Akad. Ivan Geshov 15, 1431 Sofia, Bulgaria; 3 Molecular Medicine Center, Department of Medical Chemistry and Biochemistry, Medical Faculty, Medical University of Sofia, Akad. Ivan Geshov 15, 1431 Sofia, Bulgaria; d.kachakova@medfac.mu-sofia.bg (D.L.K.-Y.); mgeorgiev@mmcbg.org (M.M.G.); 4 Department of Chemistry and Biochemistry, Medical Faculty, Medical University Sofia, Akad. Ivan Geshov 15, 1431 Sofia, Bulgaria

**Keywords:** oral squamous cell carcinoma (OSCC), whole-exome sequencing (WES), TP53 mutation, non-smoking, non-drinking (NSND) patients, young patients

## Abstract

Oral squamous cell carcinoma (OSCC) typically develops in individuals with established risk factors such as tobacco and alcohol use, yet an increasing number of cases occur in young non-smoking, non-drinking (NSND) patients. We report a case of oral tongue OSCC in a 33-year-old woman who is a never-smoker and never-drinker without identifiable environmental or local risk factors. The patient underwent surgical treatment followed by adjuvant radiotherapy and remains disease-free 15 months after therapy. Whole-exome sequencing (WES) revealed a pathogenic truncating TP53 mutation together with additional somatic alterations affecting genes involved in DNA repair, hypoxia adaptation, mitochondrial function, and epigenetic regulation. The heterogeneous mutational profile suggests branched tumor evolution and the involvement of non-classical tumorigenic pathways. This report contributes to the growing evidence that OSCC in young NSND patients represents a biologically distinct subgroup and demonstrates the value of comprehensive genomic profiling for improving understanding of tumor heterogeneity and potential molecular drivers in the absence of traditional carcinogenic exposures.

## 1. Introduction

Oral squamous cell carcinoma (OSCC) accounts for over 90% of malignant tumors in the oral cavity and remains a major global health concern despite advances in prevention and therapy [1,2]. It typically affects older adults with established risk factors such as tobacco smoking, alcohol consumption, and human papilloma virus (HPV) infection. However, an increasing number of cases have been reported in younger patients, including women, who lack such traditional exposures, representing an atypical and still poorly understood subgroup [3,4].

The genetic and molecular profile of OSCC in young, non-smoking, non-drinking (NSND) individuals appears to differ from that of conventional cases. While *TP53* mutations remain common, alterations in *RAS*, *PIK3CA*, and epigenetic pathways may also play a prominent role in tumorigenesis [5,6,7]. Risk factors like chronic trauma, poor oral hygiene, and inflammation have also been implicated [4,8]. The clinical behavior of OSCC in young patients remains controversial. Earlier studies suggested a more aggressive course, whereas more recent data indicate comparable or even improved survival when standard treatment protocols are applied [9,10,11,12,13].

Given the absence of clear environmental risk factors, genetic mechanisms have received increasing attention. Rare inherited syndromes affecting DNA repair, such as Fanconi anemia, are associated with early-onset OSCC [14], while sporadic cases in young NSND patients may share molecular features with tumors in older individuals, including TP53 mutations [15]. The most frequently somatically mutated genes in OSCC include *TP53*, *FAT1*, *CDKN2A*, *NOTCH1*, *TERT*, *CASP8*, *HRAS*, *RASA1*, *PIK3CA*, *EP300*, *CHUK*, *ZFHX4*, *KMT2D*, *ELAVL1*, and *EPHA2* [16,17,18,19,20].

Despite accumulating evidence on OSCC in young NSND individuals, these cases remain rare and insufficiently characterized, particularly with comprehensive genomic profiling. Available data on detailed clinical annotation and molecular characterization are limited, restricting integrated clinico-genomic conclusions. The present case describes tongue OSCC in a young woman without known environmental or local risk factors, with whole-exome sequencing (WES) revealing a pathogenic truncating TP53 mutation alongside heterogeneous somatic alterations. Reports combining thorough clinical characterization with WES data in young NSND OSCC patients remain scarce. This case supports the biological heterogeneity of this subgroup and suggests the involvement of non-classical tumorigenic pathways, providing further insight into the diversity of OSCC in NSND individuals and the potential role of alternative tumorigenic mechanisms.

## 2. Case Presentation

A 33-year-old woman presented to the Oral and Maxillofacial Surgery clinic in December 2024 with a persistent, non-healing lesion on the left lateral border of the tongue. The patient first noticed white mucosal changes in June 2024. She had no history of tobacco or alcohol use (she confirmed to being a never-smoker and never-drinker) and reported good oral hygiene and overall oral health, without local irritative factors such as sharp dental edges, ill-fitting prosthetic devices, or chronic trauma. The patient also denied any history of electronic cigarette use, vaping, or exposure to other nicotine delivery systems. No pain, dysphagia, dysarthria, bleeding, or weight loss were reported. HPV/p16 testing was not performed due to limited tissue availability after the histopathological analysis. We acknowledge that this represents a limitation. While HPV-associated carcinogenesis cannot be definitively excluded, oral tongue squamous cell carcinomas are generally considered less frequently associated with HPV compared to oropharyngeal tumors. Therefore, the findings are more consistent with an HPV-independent pathway, although this interpretation should be made with caution.

An incisional biopsy performed on 23 July 2024 at an outside institution was initially reported as leukoplakia without epithelial atypia. Due to persistence of the lesion, the patient sought a second opinion at our clinic. Revision of the original histological specimen demonstrated severe epithelial dysplasia with ulceration, and re-biopsy was recommended. The discrepancy between the initial and revised histopathological diagnosis is most likely attributable to sampling limitations of the initial biopsy, including insufficient depth and incomplete representation of the lesion, rather than diagnostic interpretation alone.

On clinical examination, an ulcerated lesion approximately 10 × 5 mm with elevated margins was identified on the ventral surface and lateral border of the left oral tongue in the middle third. The lesion was surrounded by a broader leukoplakic plaque measuring approximately 20 × 20 mm, more pronounced posteriorly. Palpation revealed marked perilesional induration extending beyond the visible ulceration, suggestive of an endophytic infiltrative growth pattern. No pathological cervical lymphadenopathy was detected clinically.

Repeat biopsy confirmed moderately to poorly differentiated oral squamous cell carcinoma. Preoperative ultrasonography identified a lymph node-like structure at level IV on the left side measuring 1.09 × 0.59 cm, without definitive malignant features. Magnetic resonance imaging (MRI) revealed an infiltrative contrast-enhancing lesion involving the left lateral border and ventral surface of the oral tongue, affecting the middle and partially the dorsal third. The lesion measured 25 mm in longitudinal extension with a radiologically estimated depth of invasion of 8.5 mm. Partial infiltration of intrinsic tongue musculature was noted. There was close contact without definitive invasion of the left hyoglossus muscle and the left sublingual gland, and no evidence of invasion of the sublingual fat tissue or lingual neurovascular bundle. No enlarged or suspicious cervical lymph nodes were detected at any neck level.

The case was discussed at a multidisciplinary tumor board, and surgical treatment was recommended. In December 2024, the patient underwent radical excision by left hemiglossectomy with resection of part of the floor of the mouth. The procedure included ligation of the left lingual artery and vein, ligation of the left submandibular duct, and removal of the left sublingual gland. Postoperative recovery was uneventful, and the patient was discharged in good general and local condition, tolerating oral intake, with normal wound healing and no evidence of bleeding or infection.

**Figure 1:**
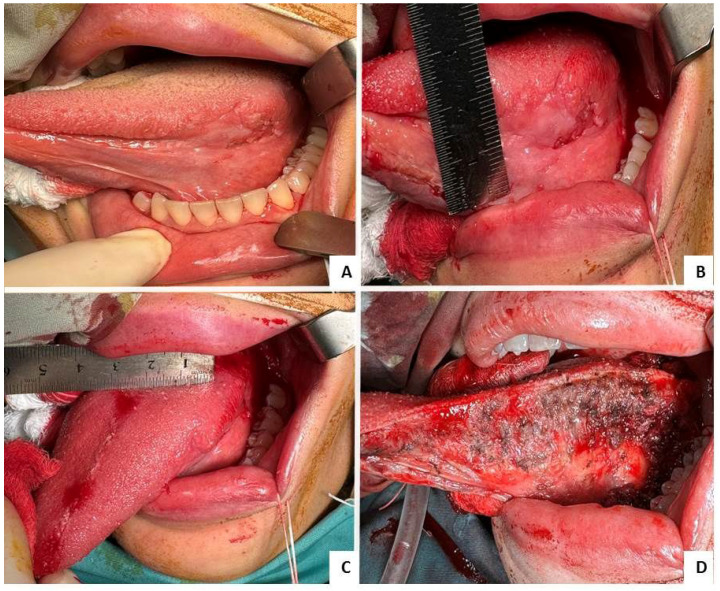
(**A**–**D**) Clinical and intraoperative images of the oral tongue squamous cell carcinoma. (**A**) Preoperative clinical presentation of the lesion on the left lateral and ventral tongue. (**B**,**C**) Intraoperative view after initial incisions outlining the tumor and planned resection margins. (**D**) Surgical defect following left hemiglossectomy with complete tumor excision.

Elective neck dissection was omitted based on the absence of clinically and radiologically suspicious lymph nodes and multidisciplinary assessment, with planned adjuvant radiotherapy addressing potential occult nodal disease. Clinical and intraoperative images illustrating the lesion and surgical resection are provided (Figure 1A–D).

Histopathological examination of the surgical specimen revealed a moderately differentiated (G2) keratinizing squamous cell carcinoma arising in leukoplakia with high-grade dysplasia, infiltrating the subepithelial connective tissue and skeletal muscle bundles. The maximum tumor diameter was 23 mm, with surface ulceration. The depth of invasion was 4 mm. The tumor demonstrated an infiltrative pattern with large and small nests (worst pattern of invasion (WPOI) grade 4) and focal lymphocytic infiltrates at the invasive front (lymphocytic response (LR) grade 2). WPOI grade 4 was defined as tumor invasion characterized by tumor islands ≥15 cells with a poorly defined infiltrative growth pattern and absence of discrete small satellite groups, according to established histopathological criteria for oral squamous cell carcinoma. LR grade 2 was defined as a moderate lymphocytic infiltrate at the tumor–stroma interface, without dense band-like or florid immune response [21]. No definite lymphovascular or perineural invasion was identified. Surgical margins were negative, with the closest margin being the deep margin at 3.5 mm. The final staging was cT2N0M0 clinically and pT2N0M0 histopathologically (AJCC 8th edition) [22].

Following surgery, the patient received adjuvant concurrent chemoradiotherapy with cisplatin. Radiotherapy was delivered using the VMAT (volumetric modulated arc therapy) technique, targeting the primary tumor bed (oral cavity) and bilateral cervical lymph node levels Ib–III, with 2.0 Gy per fraction to a total dose of 60 Gy. Additionally, bilateral cervical lymph node levels IV and V were irradiated with 1.8 Gy per fraction to a total dose of 54 Gy.

The patient reported rapid functional recovery within approximately two months after surgery, with preserved speech and oral intake. During and after radiotherapy, she developed significant treatment-related toxicity, including oral mucositis and xerostomia, followed by dysphagia, with gradual recovery over approximately 3–6 months.

Follow-up consisted of monthly clinical examinations and cervical ultrasonography, with annual PET/CT imaging. At 15 months follow-up, the patient remained disease-free, with no clinical or radiological evidence of locoregional recurrence or distant metastasis.

The patient’s medical history was notable for Hashimoto thyroiditis treated with levothyroxine (L-thyroxine 75 μg daily). She reported no known drug or food allergies.

## 3. Genetic Analysis and Molecular Findings

Peripheral blood and tumor tissue samples were obtained for germline and somatic genetic analysis respectively after receiving written informed consent for participation in our study. Genomic DNA was extracted from peripheral blood leukocytes using the Chemagic DNA isolation kit (Revvity chemagen Technologie GmbH, Baesweiler, Germany) for automated isolation with Chemagic MSM I Workstation (Revvity, Waltham, MA, USA). The tumor tissue was collected in a cryotube with RNAlater Solution (Thermo Fisher Scientific, Waltham, MA, USA) stabilizing and preventing RNA from degradation even at room temperature. The tumor tissues were transported to the lab and stored at −80 °C prior to isolation of DNA with the QIAamp DNA Mini Kit (Qiagen GmbH, Hilden, Germany) following the manufacturer’s instructions. Tumor DNA was quantified using Qubit Fluorometer (Thermo-Fisher).

Illumina DNA Prep with the Exome 2.5 Enrichment kit (Illumina Inc., San Diego, CA, USA) was used for library preparation for WES on NovaSeq 6000 (Illumina). In addition, a custom gene panel of 226 genes involved in development of hereditary oncological diseases was set, although no family history of oncological diseases was reported. We achieved mean coverage of 457.67 for the WES panel and 459.10 for the custom panel. Percent targets from the custom gene panel with coverage 100× was 95.58%; with coverage 150× was 92.07%; and with coverage 250× was 78.61%. Sequence reads were aligned against the hg19 (GRCh37) human reference genome.

The read alignment and variant calling were performed using DRAGEN Bio-IT Platform, v3 (Illumina). Binary alignment map (BAM) files were assessed for biases due to PCR duplicates, and likely duplicates were removed.

Variant annotation and filtering were performed using VarSeq 2.5 (Golden Helix Inc., Bozeman, MT, USA). Variants were filtered based on sequencing quality metrics (Variant Allele Frequency (VAF), read depth), allele frequency according to population databases, and clinical relevance (ClinVar, COSMIC), and classified according to the American College of Medical Genetics and Genomics (ACMG) guidelines [23]. The following in silico tools were used for the prediction of the effect of the point mutations (single nucleotide variants (SNVs), small deletions, insertions, indels) like: SIFT, Polyphen2, MutationTaster, MutationAssesso, FATHMM, VEST3 score, LRT score for missense mutations and GeneSplicer, MaxEntScan, NNSplice, PWM, Ada score and RF score for splice site variants.

Variants of interest were validated by Sanger sequencing using a 3500 Genetic Analyzer (Applied Biosystems™, Thermo Fisher Scientific, Waltham, MA, USA). Sanger sequencing was selectively used to validate key variants of potential clinical and biological relevance, particularly the TP53 mutation, in order to confirm variant presence and exclude potential sequencing artifacts in a single-case research setting. Validation was performed using the same DNA samples as those used for next-generation sequencing analysis.

Tumor cellularity was assessed qualitatively by histopathology and considered moderate; no formal quantitative estimation was performed, which may limit precise interpretation of variant allele frequencies.

Analysis revealed 23,759 non-synonymous SNVs across 10,609 genes and 11,602 synonymous SNVs across 6739 genes. Average on-target coverage was 303×, with 63% of the exome covered at 100–500×.

Key somatic variants of interest included a pathogenic nonsense mutation in *TP53* (c.637C>T, p.Arg213Ter, VAF 33.17%) and a missense variant of uncertain significance in TET2 (c.278G>A, p.Gly93Glu, VAF 12.94%). Sanger sequencing confirmed the somatic origin of the *TP53* mutation, as it was absent in germline DNA, and no discrepancies between next-generation sequencing and Sanger sequencing results were observed. Additional likely pathogenic variants were identified in *LAMP3*, *RDM1*, *RUFY2*, *MBD6*, *TNRC6C*, *FHOD3*, *STK25*, *ANKRD18A*, and *TFAM* (Table 1), with variant allele frequencies ranging from 5.5% to 35.2%, indicating intratumor heterogeneity. A known variant in *COL1A1* (c.992C>T, p.Ala331Val, VAF 34.87%), associated with osteogenesis imperfecta, was confirmed to be somatic.

No clinically significant pathogenic germline variants associated with hereditary cancer predisposition were detected. Heterozygous variants of uncertain significance were observed in *POT1* and *MEN1*, predicted variably as pathogenic by in silico tools. The presence of a high-allele-frequency frameshift variant in PLK5 (VAF 48.00%) may suggest a possible germline origin; however, the absence of matched normal confirmation limits definitive classification. Therefore, this variant should be interpreted with caution and considered putative until further validation.

## 4. Discussion

### 4.1. Clinical Context of OSCC in Young NSND Patients

OSCC arising in young NSND patients represents a distinct clinical and biological entity that remains insufficiently characterized. In the present case of a 33-year-old woman without established carcinogenic exposures, both the clinical presentation and molecular findings support emerging evidence that OSCC in NSND individuals, particularly women, may develop independently of traditional risk factors and may show considerable molecular heterogeneity [3,4,24].

The genetic landscape of OSCC in young NSND patients appears heterogeneous. Although TP53 mutations are among the most common alterations in OSCC overall, they may be less frequent in younger patients without tobacco or alcohol exposure [3,5]. Other molecular pathways, including alterations in PIK3CA, RAS, and epigenetic regulation, have been implicated and may contribute to carcinogenesis in the absence of classical mutagens [6,7,25]. In addition, chronic mechanical trauma, dental disease, and inflammation may promote malignant transformation, emphasizing the multifactorial nature of OSCC in this subgroup [4,8].

Management of early-stage OSCC in young patients without known risk factors generally follows established protocols; however, treatment decisions must often be individualized. Management of the clinically N0 neck remains controversial, particularly in cases with borderline depth of invasion. Although a depth of invasion ≥4 mm is commonly considered an indication for elective neck dissection, this threshold is not absolute. In the present case, the absence of clinically and radiologically suspicious cervical lymph nodes supported omission of elective neck dissection, while adjuvant radiotherapy was used to address potential occult nodal disease [26]. This approach reflects the need to balance oncologic control with treatment-related morbidity, especially in young patients with long life expectancy.

What distinguishes this case is the integration of clinical, histopathological, and genomic data in a patient without identifiable carcinogenic exposure. In contrast to many reported NSND cases, where either molecular data or clinical exclusion criteria are incomplete, this case provides further evidence for tumorigenesis independent of classical risk factors. The identification of a truncating TP53 mutation, together with multiple somatic alterations with variable allele frequencies, highlights the complexity of tumor evolution in this setting.

HPV testing was not performed in this case due to limited availability of residual tissue after diagnostic and histopathological processing, which represents a limitation. While HPV-associated carcinogenesis cannot be definitively excluded, the anatomical location of the tumor in the oral tongue does not strongly support a typical HPV-driven presentation. Therefore, the findings are more consistent with an HPV-independent pathway, although this interpretation should be made with caution. Inclusion of HPV testing in future cases would allow more precise etiological characterization.

### 4.2. Key Somatic Driver: TP53 Mutation

Comprehensive genomic profiling identified a somatic truncating mutation in TP53 (c.637C>T, p.Arg213Ter) with a variant allele frequency of 33.17%. This nonsense mutation results in premature termination of the p53 protein and loss of tumor suppressor function, in contrast to dominant-negative missense variants [27,28,29,30]. Loss-of-function TP53 alterations are associated with impaired cell cycle control, genomic instability, and potentially reduced sensitivity to radiotherapy and systemic therapy [29,30].

The relatively high variant allele frequency suggests that the TP53 mutation occurred early in tumorigenesis and likely represents an initiating event. In addition to TP53, the tumor harbored somatic variants affecting DNA repair (RDM1), hypoxia response (LAMP3), mitochondrial regulation (TFAM), and other pathways, indicating the involvement of multiple biological processes in tumor progression.

### 4.3. Additional Somatic Alterations and Tumor Heterogeneity

The tumor demonstrated a heterogeneous mutational profile, including frameshift and nonsense variants in *LAMP3*, *RDM1*, *RUFY2*, *MBD6*, *TNRC6C*, *FHOD3*, *STK25*, *ANKRD18A*, and *TFAM* (Supplementary Table S1, Supplementary Text S1). Variants in LAMP3 and RDM1 showed relatively higher allele frequencies, suggesting potential biological relevance.

LAMP3 has been associated with hypoxia adaptation and metastatic processes [31,32], while RDM1 is involved in DNA repair and homologous recombination [33,34]. TFAM alterations may affect mitochondrial function and cellular metabolism. Variants in MBD6 and TNRC6C suggest the additional involvement of epigenetic and post-transcriptional regulatory mechanisms. However, their functional roles in OSCC remain unclear and require further validation.

Interpretation of variant allele frequencies is limited by the lack of quantitative tumor cellularity assessment. Since tumor purity directly influences VAF, distinction between clonal and subclonal variants remains approximate. Therefore, conclusions regarding clonal architecture should be interpreted with caution.

Although several alterations appear potentially relevant, their functional significance in OSCC remains uncertain and should be considered hypothesis-generating, particularly for genes such as *LAMP3*, *RDM1*, and *TFAM*.

### 4.4. Implications for Tumorigenesis in NSND Patients

Taken together, the findings support a model in which tumor development is driven by combined defects in DNA repair, microenvironmental adaptation, and metabolic regulation rather than a single dominant oncogenic pathway. This perspective may be particularly relevant in NSND patients, where tumorigenesis is more likely driven by endogenous molecular dysregulation rather than exogenous mutagenic exposure.

The coexistence of somatic alterations with varying allele frequencies suggests a branched evolutionary pattern rather than linear mutation accumulation. However, this interpretation is limited by the absence of precise tumor cellularity estimation; thus, the proposed evolutionary model remains hypothetical.

Most additional variants identified in this case are likely passenger events reflecting intratumor heterogeneity rather than established driver mutations. A detailed summary of all variants, including gene annotation, VAF, predicted impact, and putative function, is provided in Supplementary Table S1 and Supplementary Text S1.

Alterations in genes involved in hypoxia response (LAMP3), DNA repair (RDM1), and epigenetic regulation (TET2) further support the involvement of non-classical oncogenic pathways. Although their functional relevance remains to be clarified, these findings highlight the need for further genomic and translational studies in young NSND patients.

### 4.5. Comparative Genomic Context with Public Datasets

To contextualize the molecular findings, we compared them with publicly available genomic datasets, including The Cancer Genome Atlas (TCGA) head and neck squamous cell carcinoma (HNSCC) cohort. In TCGA HNSCC, *TP53* is the most frequently altered gene, occurring in approximately 70–80% of cases, followed by recurrent alterations in *CDKN2A*, *FAT1*, *NOTCH1*, and *PIK3CA*, which define the canonical driver landscape [35,36].

Previous studies also report distinct mutational patterns in OSCC from non-smoking, non-drinking (NSND) patients, including differences in mutation burden and gene alteration frequencies compared with exposure-associated cases [37].

In the present case, the tumor exhibits a heterogeneous mutational profile characterized by a truncating TP53 mutation together with additional low-frequency somatic alterations affecting DNA repair, mitochondrial function, hypoxia response, and epigenetic regulation. Overall, this pattern suggests increased genomic heterogeneity and a more context-dependent tumor evolution in NSND-associated OSCC compared with classical tobacco-related tumors.

### 4.6. Clinical Implications

From a clinical perspective, this case highlights several important points. First, OSCC in young NSND individuals underscores the need for a low threshold for biopsy of persistent oral lesions, even when initial histology appears benign. Second, the discrepancy between initial diagnosis and final pathology emphasizes the importance of histological revision and re-biopsy in persistent lesions. This highlights the importance of adequate biopsy sampling in suspicious oral lesions, as superficial or non-representative samples may lead to underdiagnosis. Third, comprehensive genomic profiling provides additional biological insight, although it does not yet directly influence standard treatment.

The patient experienced treatment-related toxicity, including mucositis, xerostomia, and dysphagia, which gradually improved over several months, emphasizing the importance of supportive care and rehabilitation [38]. Toxicity was graded according to CTCAE version 5.0 [39]. The patient developed grade 2–3 oral mucositis and grade 1–2 xerostomia, with peak severity during weeks 3–5 of therapy. Dysphagia was graded as CTCAE grade 2 during the acute phase.

Overall, this case adds to the evidence suggesting that OSCC in young NSND patients may represent a biologically distinct subgroup characterized by TP53-driven tumorigenesis and additional molecular heterogeneity. These findings may contribute to future risk stratification and personalized approaches.

From a prognostic perspective, TP53 loss-of-function mutations are associated with genomic instability and potentially reduced response to therapy [40]. In this case, TP53 status did not alter treatment decisions but may have future implications for molecular stratification in NSND OSCC.

### 4.7. Limitations

Key limitations include the absence of HPV/p16 testing, which prevents definitive exclusion of HPV-associated carcinogenesis; the presence of variants of uncertain significance requiring functional validation; and the single-case design, limiting generalizability. Histopathological images, including material from the initial biopsy, were not available for inclusion, which limits the visual documentation of the diagnostic discrepancy.

An additional limitation is the lack of quantitative tumor cellularity assessment, which may affect interpretation of variant allele frequencies and clonal structure.

### 4.8. Future Directions

Future research should include larger cohorts of young NSND patients, functional validation of candidate variants—particularly those involved in DNA repair, hypoxia adaptation, and mitochondrial regulation—and investigation of clonal evolution and therapeutic implications. These studies may support development of personalized treatment strategies for this subgroup.

## 5. Conclusions

In conclusion, this case highlights OSCC in a young NSND woman as a rare but clinically significant presentation. Somatic TP53 mutation emerged as a central driver in the absence of traditional carcinogenic exposures, while additional alterations in genes such as LAMP3 and RDM1 suggest that alternative molecular pathways may contribute to tumorigenesis in this subgroup. This report adds to growing evidence that OSCC in young NSND patients represents a biologically distinct entity and demonstrates the value of comprehensive genomic profiling in uncovering non-classical tumorigenic mechanisms, with potential implications for risk stratification and personalized therapeutic approaches.

## Figures and Tables

**Table 1:** Somatic mutations of interest found in analyzed tumor sample.

Gene	Variant	VAF (%)	Prediction
*TP53*	c.637C>T (p.Arg213Ter)	33.17	Pathogenic
*TET2*	c.278G>A (p.Gly93Glu)	12.94	Variant of uncertain significance
*COL1A1*	c.992C>T (p.Ala331Val)	34.87	Likely pathogenic
*LAMP3*	c.995_1014del (p.Ala332Valfs*4)	35.19	Likely pathogenic
*RUFY2*	c.352delA (p.Met118Trpfs*8)	5.51	Likely pathogenic
*MBD6*	c.2345dupG (p.Ala783Serfs*10)	5.81	Likely pathogenic
*RDM1*	c.334delT (p.Ser112Profs*46)	28.61	Likely pathogenic
*TNRC6C*	c.1325C>G (p.Ser442Ter)	5.54	Likely pathogenic
*FHOD3*	c.1381_1382delAG (p.Arg461Alafs*48)	5.58	Likely pathogenic
*STK25*	c.1123C>T (p.Gln375Ter)	11.04	Likely pathogenic
*ANKRD18A*	c.2118-1G>C	11.00	Likely pathogenic
*TFAM*	c.441delA (p.Glu148Serfs*2)	9.94	Likely pathogenic

## Data Availability

The data that support the findings of this study are available on request from the corresponding author. The data is not publicly available due to privacy and ethical restrictions.

## Supplementary Materials

The following supporting information can be downloaded at: https://www.mdpi.com/article/10.3390/reports9020130/s1, Supplementary Table S1. Somatic variants identified in the tumor sample. Text S1. Extended interpretation of additional variants.

## Abbreviations

The following abbreviations are used in this manuscript:

OSCC	Oral squamous cell carcinoma
HPV	Human papilloma virus
NSND	Non-smoking, non-drinking
WES	Whole exome sequencing
MRI	Magnetic resonance imaging
WPOI	Worst pattern of invasion
BAM	Binary alignment map
SNVs	Single nucleotide variants
VAF	Variant Allele Frequency
CTCAE	Common Terminology Criteria for Adverse Events
SCC	Squamous cell carcinoma
HNSCC	Head and neck squamous cell carcinoma
