# Supplementary material for: Early-Onset Oral Tongue Squamous Cell Carcinoma in the Absence of Traditional Risk Factors: A Case Report with Whole-Exome Sequencing Analysis
Source: Reports (MDPI). 2026 Apr 24;9(2):130. doi: 10.3390/reports9020130 (PMC13214632; doi:10.3390/reports9020130)
Supplement: Supplementary file 1 [file reports-09-00130-s001.zip › reports-4259396-supplementary.pdf]

Supplementary Table S1. Somatic variants identified in the tumor sample

Gene	Variant (cDNA / protein)	Variant type	VAF (%)	Classification	Putative role
**TP53**	**c.637C>T (p.Arg213Ter)**	**Nonsense**	**33.17**	**Pathogenic**	**Core tumor suppressor**
**TET2**	**c.278G>A (p.Gly93Glu)**	**Missense**	**12.94**	**VUS**	**Epigenetic regulation**
**COL1A1**	**c.992C>T (p.Ala331Val)**	**Missense**	**34.87**	**Likely pathogenic**	**ECM remodeling**
**LAMP3**	**c.995_1014del (p.Ala332Valfs*4)**	**Frameshift**	**35.19**	**Likely pathogenic**	**Hypoxia response, metastasis**
**RUFY2**	**c.352delA (p.Met118Trpfs*8)**	**Frameshift**	**5.51**	**Likely pathogenic**	**Vesicle trafficking, autophagy**
**MBD6**	**c.2345dupG (p.Ala783Serfs*10)**	**Frameshift**	**5.81**	**Likely pathogenic**	**Chromatin regulation**
**RDM1**	**c.334delT (p.Ser112Profs*46)**	**Frameshift**	**28.61**	**Likely pathogenic**	**DNA repair**
**TNRC6C**	**c.1325C>G (p.Ser442Ter)**	**Nonsense**	**5.54**	**Likely pathogenic**	**miRNA-mediated silencing**
**FHOD3**	**c.1381_1382delAG (p.Arg461Alafs*48)**	**Frameshift**	**5.58**	**Likely pathogenic**	**Cytoskeleton organization**

**STK25**	**c.1123C>T (p.Gln375Ter)**	**Nonsense**	**11.04**	**Likely pathogenic**	**Hippo signaling pathway**
**ANKRD18A**	**c.2118-1G>C**	**Splice-site**	**11.00**	**Likely pathogenic**	**Transcriptional regulation**
**TFAM**	**c.441delA (p.Glu148Serfs*2)**	**Frameshift**	**9.94**	**Likely pathogenic**	**Mitochondrial function**
**PLK5**	**c.672dupG (p.Arg225Alafs*38)**	**Frameshift**	**48.00**	**Likely germline (unconfirmed)**	**Cell cycle regulation**

Supplementary Text S1. Extended interpretation of additional variants: In addition to the pathogenic *TP53* mutation, comprehensive genomic profiling of the tumor revealed multiple somatic variants with potential functional relevance (Table 1).

TET2 (c.278G>A, p.Gly93Glu, VAF 12.94%) is a missense variant of uncertain significance (VUS) involved in epigenetic regulation. Although its pathogenicity in oral squamous cell carcinoma (OSCC) is not established, *TET2* alterations have been implicated in aberrant DNA methylation and transcriptional dysregulation in various cancers.

COL1A1 (c.992C>T, p.Ala331Val, VAF 34.87%) is a missense variant classified as likely pathogenic. COL1A1 encodes a major component of the extracellular matrix (ECM), and alterations may influence tumor-stroma interactions and facilitate invasive behavior. Sanger sequencing confirmed the somatic origin of this variant.

LAMP3 (c.995_1014del, p.Ala332Valfs*4, VAF 35.19%) is a frameshift variant predicted to disrupt protein function. *LAMP3* has been associated with hypoxia adaptation, tumor cell survival, and metastatic potential in squamous cell carcinomas.

Other frameshift and nonsense variants with lower variant allele frequencies include:

RUFY2 (c.352delA, p.Met118Trpfs*8, VAF 5.51%), involved in vesicle trafficking and autophagy;

MBD6 (c.2345dupG, p.Ala783Serfs*10, VAF 5.81%), a chromatin regulator potentially affecting transcription;

RDM1 (c.334delT, p.Ser112Profs*46, VAF 28.61%), implicated in DNA repair and homologous recombination;

TNRC6C (c.1325C>G, p.Ser442Ter, VAF 5.54%), a component of miRNA-mediated gene silencing;

FHOD3 (c.1381_1382delAG, p.Arg461Alafs*48, VAF 5.58%), regulating cytoskeleton dynamics;

STK25 (c.1123C>T, p.Gln375Ter, VAF 11.04%), involved in Hippo signaling;

ANKRD18A (c.2118-1G>C, VAF 11.00%), a predicted splice-site variant with potential transcriptional regulatory impact;

TFAM (c.441delA, p.Glu148Serfs*2, VAF 9.94%), essential for mitochondrial transcription and genome maintenance.

A germline frameshift variant in *PLK5* (c.672dupG, p.Arg225Alafs*38, VAF 48%) was also detected. Although Sanger validation was not performed, the high VAF suggests a potential germline origin. *PLK5* is implicated in cell cycle regulation and may contribute to tumor suppressive pathways if disrupted.

The range of variant allele frequencies observed across these genes indicates intratumoral heterogeneity. Collectively, these alterations may contribute to diverse oncogenic mechanisms, including epigenetic dysregulation, DNA repair deficiency, metabolic adaptation, cytoskeletal remodeling, and dysregulated signaling pathways. Functional validation of these variants in OSCC is warranted to determine their potential role in tumorigenesis and as possible therapeutic targets.
